# Coronary CT Angiography in Heavily Calcified Coronary Arteries: Improvement of Coronary Lumen Visualization and Coronary Stenosis Assessment With Image Postprocessing Methods

**DOI:** 10.1097/MD.0000000000002148

**Published:** 2015-12-07

**Authors:** Zhonghua Sun, Curtise K.C. Ng, Lei Xu, Zhanming Fan, Jing Lei

**Affiliations:** From the Department of Medical Radiation Sciences, Curtin University, Perth, Western Australia, Australia (ZS, CKCN), Department of Radiology, Beijing Anzhen Hospital, Capital Medical University, Beijing, China (LX, ZF), and Department of Medical Imaging, First Affiliated Hospital of Kunming Medical University, Yunnan, China (JL).

## Abstract

To compare the diagnostic value of coronary CT angiography (CCTA) with use of 2 image postprocessing methods (CCTA_S) and (CCTA_OS) and original data (CCTA_O) for the assessment of heavily calcified plaques.

Fifty patients (41 men, 9 women; mean age 61.9 years ± 9.1) with suspected coronary artery disease who underwent CCTA and invasive coronary angiography (ICA) examinations were included in the study. Image data were postprocessed with “sharpen” and smooth reconstruction algorithms in comparison with the original data without undergoing any image postprocessing to determine the effects on suppressing blooming artifacts due to heavy calcification in the coronary arteries. Minimal lumen diameter and degree of stenosis were measured and compared between CCTA_S, CCTA_OS, and CCTA_O with ICA as the reference method. The area under the curve (AUC) by receiver-operating characteristic curve analysis (ROC) was also compared among these 3 CCTA techniques.

On a per-vessel assessment, the sensitivity, specificity, positive predictive value and negative predictive value, and 95% confidence interval (CI) were 100% (95% CI: 89%, 100%), 33% (95% CI: 22%, 45%), 41% (95% CI: 30%, 53%), 100% (95% CI: 85%, 100%) for CCTA_O, 94% (95% CI: 79%, 99%), 66% (95% CI: 54%, 77%), 57% (95% CI: 43%, 70%), and 95% (95% CI: 85%, 99%) for CCTA_S, 94% (95% CI: 79%, 99%), 44% (95% CI: 32%, 57%), 44% (95% CI: 32%, 57%), and 97% (95% CI: 79%, 99%) for CCTA_OS, respectively. The AUC by ROC curve analysis for CCTA_S showed significant improvement for detection of >50% coronary stenosis in left anterior descending coronary artery compared to that of CCTA_OS and CCTA_O methods (*P* < 0.05), with no significance differences for detection of coronary stenosis in the left circumflex and right coronary arteries (*P* > 0.05).

CCTA with “sharpen” reconstruction reduces blooming artifacts from heavy calcification, thus, leading to significant improvement of specificity and positive predictive value of CCTA in patients with heavily calcified plaques. However, specificity is still moderate and additional functional imaging may be needed.

## INTRODUCTION

The diagnostic performance of coronary CT angiography (CCTA) is widely known to be affected in the presence of severely calcified plaques in the coronary arteries due to blooming artifacts resulting from heavy calcification, thus, leading to high false positive rates of coronary lumen stenosis.^1–5^ The specificity of CCTA still remains limited, despite improvements in CT technology, since extensive calcification in the coronary artery segments is often associated with uninterpretable analysis or results in overestimation of stenosis.^6^ This limitation has been addressed by 2 approaches: use of iterative reconstruction (IR) techniques in the CT raw data for reduction of image noise while improving assessment of calcification in the coronary arteries; and application of image postprocessing algorithms for suppression of the effect of heavy calcification on the coronary lumen visualization.^7,8^

In recent years, the use of IR has been mainly focused on lowering radiation dose of CCTA in comparison with the conventional approach of filtered back projection (FBP).^9–12^ A recent systematic review has shown that CCTA using IR technique results in significantly lower radiation dose with improvement of image quality when compared to CCTA using FBP.^7^ Over the last few years, there has been an increasing interest in using IR technique in the imaging analysis of morphological characteristics of coronary plaques, such as plaque volume or plaque composition.^13–19^ However, findings about the effect of IR on quantitative assessment of calcified plaques are inconclusive. Studies in vitro and in vivo investigating the effects of different IR techniques on calcium score analysis showed that CCTA with use of IR significantly reduced calcium volume and calcium scores,^13–17^ leading to improved diagnostic value of CCTA in heavily calcified coronary arteries,^18^ while others reported no significant effects on measurements of calcified plaque volume using IR compared to FBP.^19^

We have recently reported our experience of using different image postprocessing algorithms in the assessment of CCTA images with high calcium scores, and showed the improved visualization of coronary artery lumen with use of image postprocessing “sharpen” algorithm.^8^ Due to limited information on the effects of image processing methods on the calcified coronary plaques,^8,20^ the purpose of this study is to apply the appropriate image postprocessing reconstruction derived from our preliminary findings to a group of patients with heavily calcified coronary plaques. We hypothesized that diagnostic value of CCTA, in particular, the specificity and positive predictive value (PPV) might be improved in the evaluation of heavily calcified plaques with use of the appropriate image processing method.

## METHODS

### Patient Data

This retrospective study reviewed 100 consecutive patients who were referred for the assessment of suspected coronary artery disease (CAD) with CCTA on ≥64-slice CT scanners between March and August 2014. Eligible patients included adults with at least one coronary artery segment with calcified plaques identified on CCTA, with invasive coronary angiography (ICA) performed as the reference method for diagnosis of coronary artery stenosis. Patients were not eligible if they had any of the following conditions: known allergy for contrast media, prior history of coronary artery bypass surgery, prior coronary stenting, impaired renal function with serum creatinine level >1.5 mg/dL, or unable to control heart rate less than 65 beats per minute despite use of beta-blockers. Fifty patients were excluded due to the following reasons: coronary plaques other than calcified type (mixed or noncalcified plaques) (n = 15), ICA was not available for comparison (n = 30), and coronary stenting (n = 5). The final cohort consisted of 50 patients with patient characteristics shown in Table 1.

**TABLE 1:**
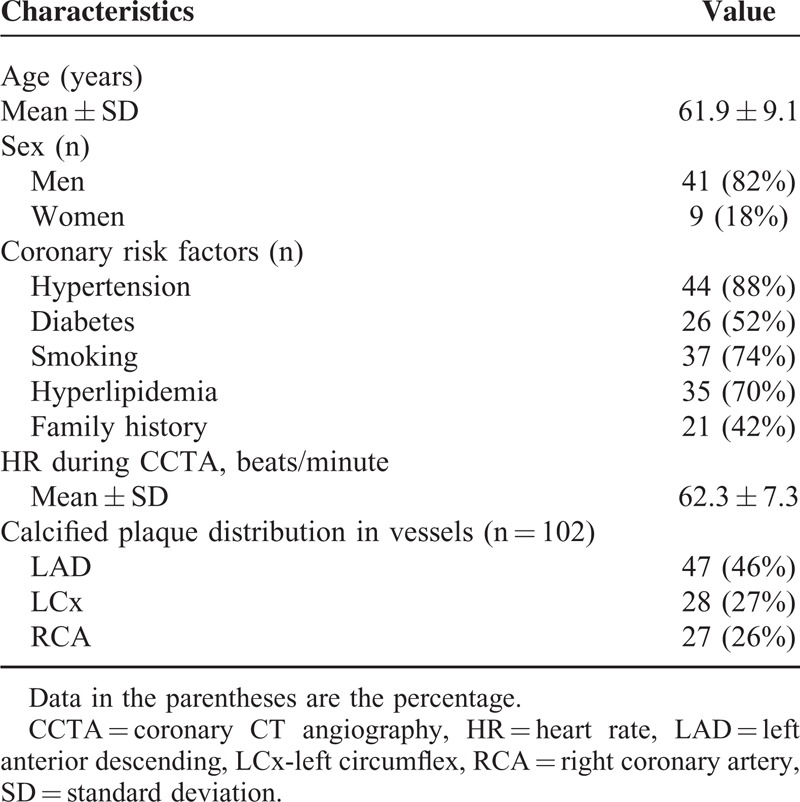
Patient Characteristics (n = 50)

CCTA image acquisition was obtained as part of clinical cardiac CT examinations; therefore, the need for institutional review board approval was waived by the local ethics committee for this study. No informed consent was obtained from the patients, given the retrospective nature of the study.

### CCTA Scanning Protocol

The patients were scanned with a first-generation dual-source CT (Somatom Definition Flash, Siemens Healthcare, Forchheim, Germany), or a second-generation dual-source CT (Somatom Definition Flash); or 640-slice CT (Toshiba Aquilion ONE, Toshiba, Otawara, Japan), with corresponding 20 patients (40%) for 64-slice CT, 15 patients (30%) for 128-slice CT, and 15 patients (30%) for 640-slice CT, respectively. The imaging protocol has been previously described in detail.^21^ Intravenous contrast medium (Iopromide 370, Bayer Schering Pharma, Guangzhou, China) was administered during each scan with details described in our previous study.^21^ Images were reconstructed with a slice thickness of 0.6 to 0.75 mm and a reconstruction interval of 0.5 to 0.6 mm for the first- and second-generation dual-source CT, slice thickness of 0.5 mm and reconstruction interval of 0.25 mm for 640-slice CT, respectively.

Although data were acquired with different CT scanners, our previous study did not show any significant difference in the diagnostic value between different multislice CT vendors.^21^ Therefore, this study analysis is considered comparable between the different vendors.

### Image Postprocessing

Original 2D axial CT images in DICOM (digital imaging and communications in medicine) and curved planar reformatted images were processed by a Windows 7 64-bit workstation with open source software ImageJ v 1.49b (National Institutes of Health, Bethesda, MD, USA) using common spatial domain processing approaches.^22^ Details of the image processing algorithms used in this study have been described in our recent report. The ImageJ software provides default functions to perform the image processing conveniently. It also allows users to conduct nondefault image processing techniques with flexibility. For example, the default ImageJ “sharpen” function shows the most effective outcomes and it is actually a function to carry out the convolution filtering with the following kernel (convolution matrix/mask).

-1 -1 -1

-1 12 -1

-1 -1 -1

The use of ImageJ “sharpen” function has been proved to effectively suppress artifacts due to heavy calcification, thus, this image postprocessing algorithm was selected for evaluation of CCTA images in this study. In addition, another commonly used approach, smoothed image subtracted from original data and the outcome integrated with the original image subsequently was also included in the image analysis. The default ImageJ function was used to smooth images in this approach. It is again based on the convolution filtering technique with the use of the following kernel.

1 1 1

1 1 1

1 1 1

This technique is more commonly known as the 3 × 3 mean filtering. The image subtraction process was handled by the default ImageJ “image calculator” function.^8^ In summary, 3 different imaging datasets were analyzed in 50 patients, namely: CCTA original data (CCTA-O), CCTA with use of “sharpen” algorithm (CCTA-S), and CCTA original data integrated with the outcome of smoothed image subtracted from the original (CCTA-OS).

### CCTA Measurements

Data were transferred to a separate workstation equipped with Analyze V 11.0 (AnalyzeDirect, Inc., Lexana, KS) for image postprocessing and analysis. The minimal lumen diameter (MLD) of three main coronary arteries (including LAD—left anterior descending, LCx—left circumflex, and RCA—right coronary artery) was measured using the same approach as described in our previous study.^21^ Measurements were performed by 2 researchers (with 5 and 10 more years of experience in CCTA, respectively) who were unaware of the results of ICA. Two reviewers assessed CCTA and ICA images separately with an interval of 2 weeks. Any discrepancy was resolved by a reading session in which both reviewers read images together to reach a consensus. Three consecutive measurements of the MLD at each coronary lesion were obtained, and the mean value was averaged to avoid intraobserver disagreement.

The percentage of coronary lumen reduction (stenosis) was calculated using the following formula: 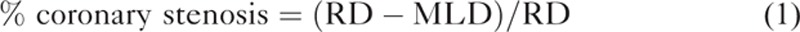


RD refers to the reference normal diameter of coronary artery. The degree of coronary lumen stenosis was determined significant when the percentage of reduction was measured >50%.^23^ When there were multiple calcified plaques present in the coronary arteries, measurements were performed at the most severely calcified lesions as this study only focused on the heavily calcified plaques.

### Invasive Coronary Angiography

Invasive coronary angiography (ICA) was performed by femoral or radial approach. The MLD was measured in projections showing the most severe narrowing of three main coronary arteries (LAD, LCx, and RCA) by a radiologist with more than 15 years of experience in cardiac imaging. Similarly, 3 consecutive measurements of the MLD within the same lesion were obtained, and the mean value was averaged.

### Statistical Analysis

Statistical analyses were performed using SPSS 21.0 (SPSS, Inc, Chicago, IL). All continuous variables were expressed as the mean ± standard deviation, while categorical variables were presented as frequencies or percentages. Sensitivity, specificity, positive predictive value (PPV), and negative predictive value (NPV) for the detection of significant stenosis (>50%) on CCTA were calculated for individual three main coronary arteries with ICA as the gold standard. Receiver-operating characteristic (ROC) curve analysis was used to evaluate the diagnostic value of CCTA using different image postprocessing approaches in the measurement of MLD and detection of coronary stenosis compared to ICA. The areas under the ROC curves (AUCs) were compared among these three methods. A difference with *P*-value of <0.05 was considered statistically significant.

## RESULTS

A total of 102 calcified coronary arteries from 50 patients were detected and analyzed. Calcified plaques were found in 47 LAD, 28 LCx, and 27 RCA arterial branches, respectively. ICA showed coronary stenosis greater than 50% in 26 (52%) patients, with a total of 33 coronary arteries. More than half of calcified plaques (19 out of 33 lesions, 57%) were found to involve the LAD. Twenty patients had 1-vessel disease, 5 patients had 2-vessel disease, and the remaining 1 patient had 3-vessel disease.

We randomly selected 20 cases (60 vessels) for interobserver variability and the comparison of MLD measurements between CCTA and ICA. The 2 readers blindly measured MLD and RD on these techniques individually, with results showing high correlation between these values performed by two observers (r = 0.82–0.94, *P* < 0.05).

The sensitivity, specificity, PPV and NPV and 95% confidence interval (CI) for CCTA_O, CCTA_S, and CCTA_OS in individual and all of the 3 coronary arteries are presented in Table 2. By using image “sharpen” reconstruction (CCTA_S), there was a significant improvement in specificity and PPV on a per-vessel level for the detection of significant stenosis when compared to the CCTA_OS and CCTA_O. No significant changes were found with use of smooth reconstruction (CCTA_OS), although the specificity was slightly improved when compared to the original data as shown in Table 2.

**TABLE 2:**
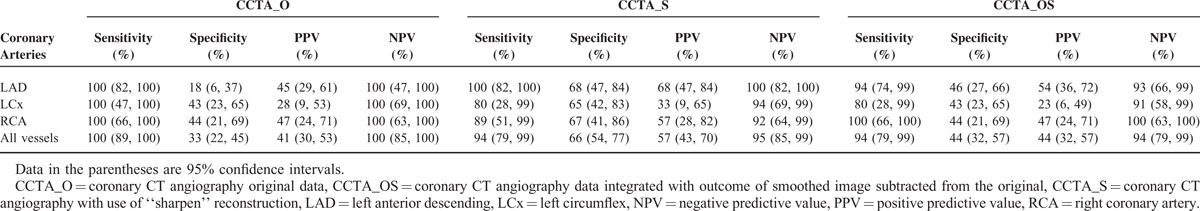
Diagnostic Value of CCTA With Use of Different Image Processing Approaches When Compared to Invasive Coronary Angiography

**FIGURE 1:**
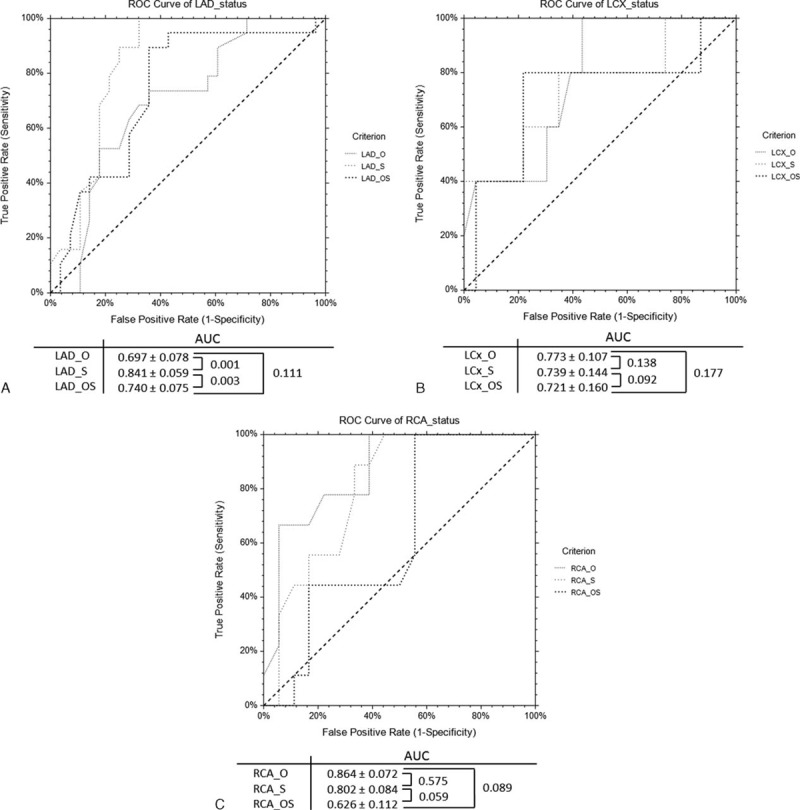
AUCs by receiver-operating characteristic curve analysis demonstrate the diagnostic performance of coronary CT angiography (CCTA) original (CCTA_O), with use of image “sharpen” (CCTA_S), and smooth algorithms (CCTA_OS) in the detection of significant coronary stenosis when compared to invasive coronary angiography at left anterior descending (LAD), left circumflex (LCx), and right coronary artery (RCA) (A–C).

**FIGURE 2:**
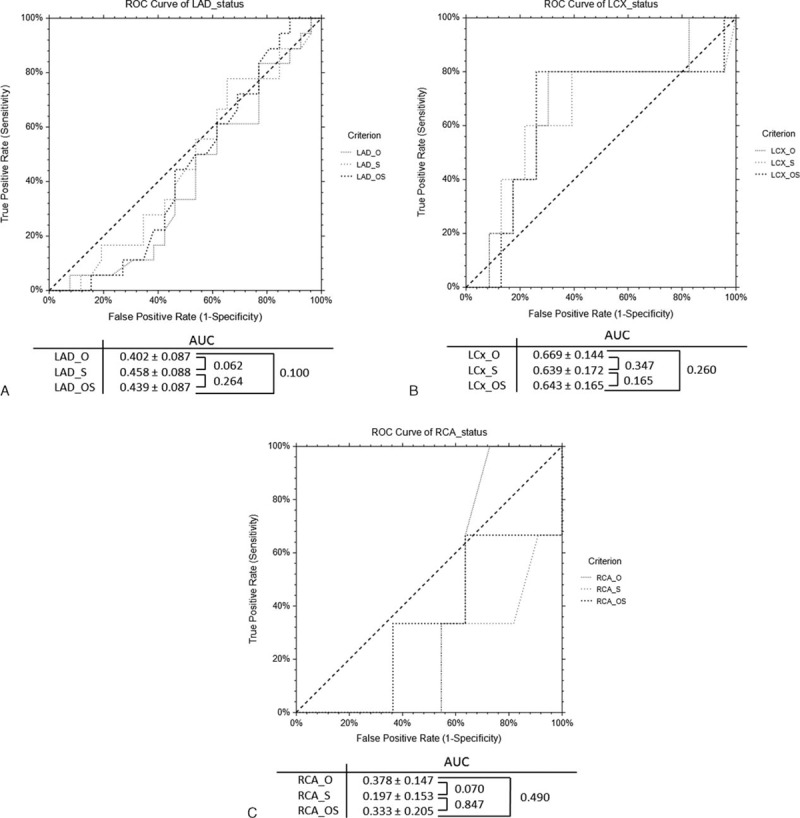
AUCs by receiver-operating characteristic curve analysis demonstrate the diagnostic performance of coronary CT angiography (CCTA) original (CCTA_O), with use of image “sharpen” (CCTA_S), and smooth algorithms (CCTA_OS) in the measurements of minimal lumen diameter (MLD) when compared to invasive coronary angiography at LAD, LCx, and RCA (A–C).

The AUCs by ROC curve analysis for CCTA_S demonstrated significant improvement for detection of >50% coronary stenosis in LAD compared to other 2 methods (*P* < 0.05) (Fig. 1A), with no significant difference for detection of significant coronary stenosis in LCx among the 3 methods (*P* = 0.09–0.17) (Fig. 1B). The AUCs by ROC curve analysis for CCTA detection of 50% coronary stenosis in RCA showed no improvement with use of image postprocessing algorithms with CCTA_OS having the lowest AUCs, although this did not reach statistical significance (*P* = 0.05–0.57) (Fig. 1C). In contrast, the AUCs by ROC analysis for CCTA assessment of MLD indicated very low values in LAD and RCA, with better performance of CCTA in LCx among the 3 methods, although there was no significant difference between the comparisons (Fig. 2).

**FIGURE 3:**
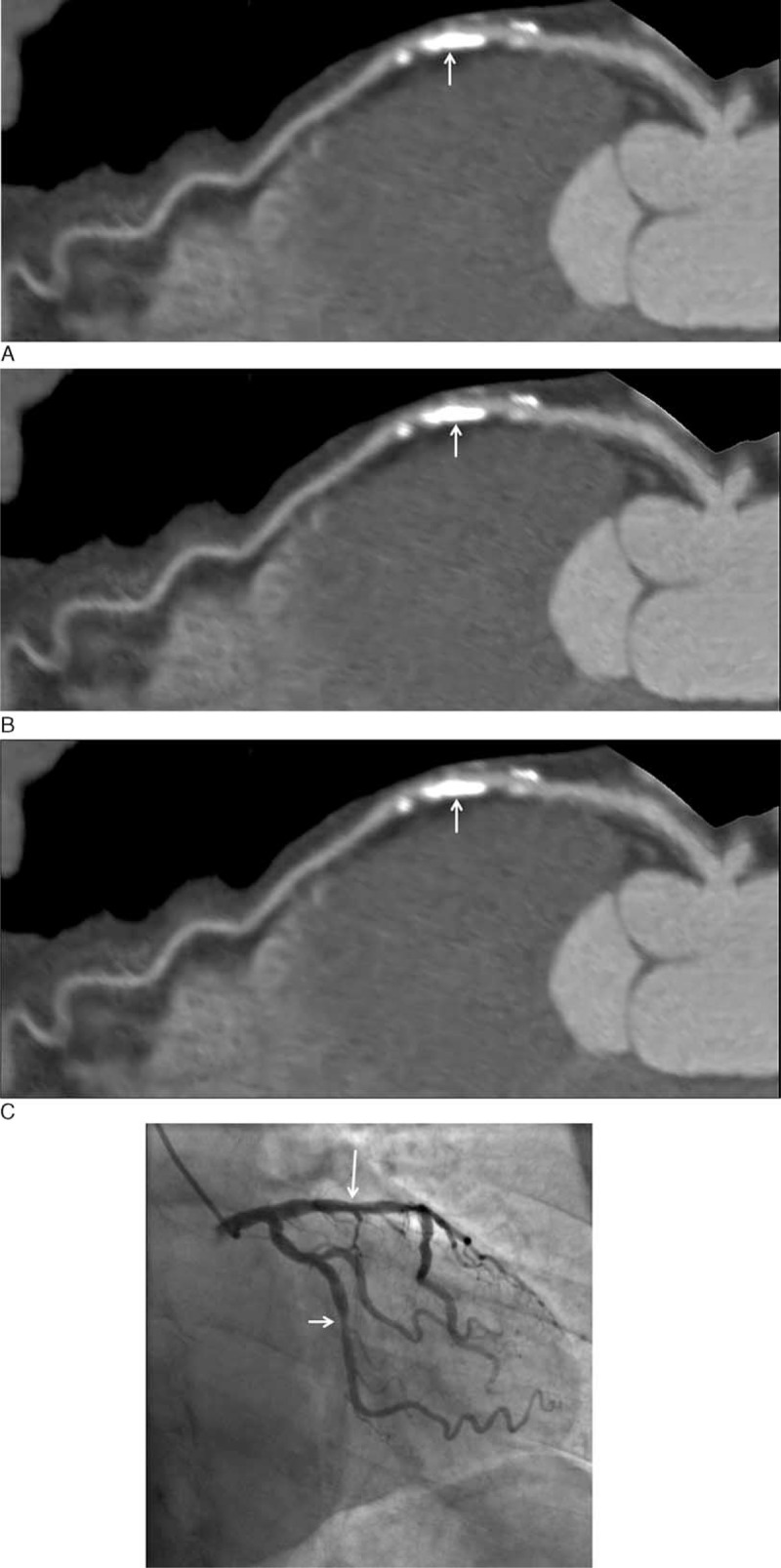
(A) Curved planar reformatted coronary CT angiography (CCTA) shows multiple calcified plaques at the left anterior descending coronary artery (LAD) in a 59-year-old man. CCTA original data (CCTA_O) shows 69% stenosis in LAD due to the heavily calcified plaque, while CCTA with “sharpen” algorithm (CCTA_S) demonstrates 45% stenosis, and 68% stenosis as shown with CCTA with original data integrated with outcome of smoothed image subtracted from the original (CCTA_OS) (A–C). Invasive coronary angiography confirms mild stenosis of 28% at LAD and 44% stenosis at LCx (D). Long and short arrows refer to the stenosis at LAD and LCx, respectively.

**FIGURE 4:**
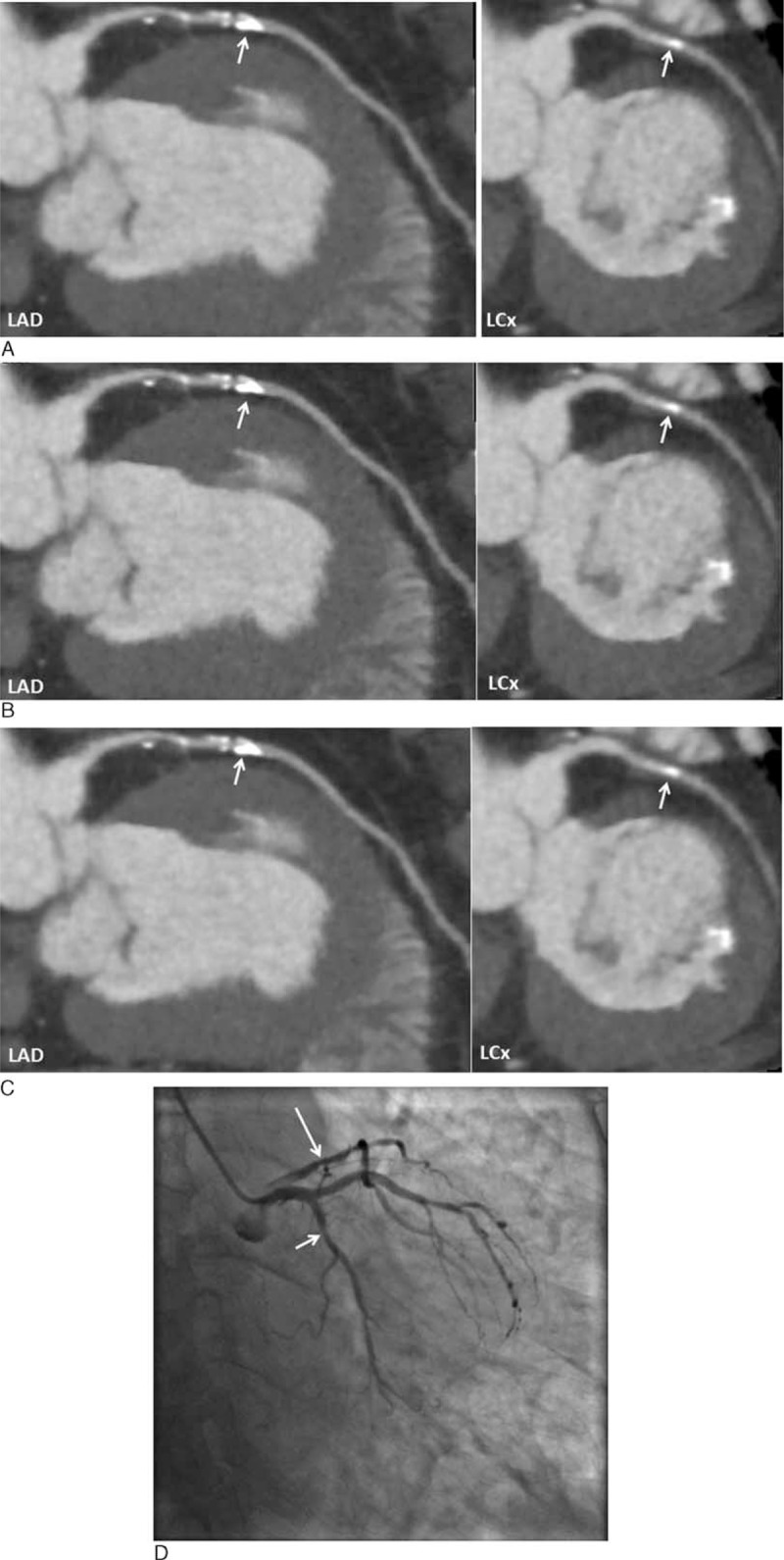
Curved planar reformatted coronary CT angiography (CCTA) shows multiple calcified plaques at the left anterior descending coronary artery (LAD) and left circumflex coronary artery (LCx) in a 75-year-old man. Significant stenosis due to severe calcification was noticed at LAD and LCx with 88% and 68%, 73% and 70%, and 87% and 74% stenoses, corresponding to CCTA-O, CCTA-S, and CCTA_OS, respectively (A–C). Invasive coronary angiography confirms 28% and 45% stenoses at LAD and LCx (D). Long and short arrows refer to the stenosis at LAD and LCx, respectively.

Figure 3 shows CCTA analysis in a patient with severely calcified plaques in LAD with improvement of coronary lumen visualization and degree of stenosis by CCTA_S when compared to the other 2 methods. Figure 4 demonstrates another case with heavy calcification in LAD and LCx with no improvement in both lumen and coronary stenosis by these techniques compared to ICA.

## DISCUSSION

Findings of this study show that the use of image postprocessing method, the “sharpen” reconstruction algorithm significantly improves the specificity and PPV for CCTA detection of coronary stenosis in patients with heavily calcified plaques. These improvements are most likely due to the suppression of blooming artifacts resulting from the heavy calcification in the coronary arteries, thus, leading to better coronary lumen visualization and assessment of degree of stenosis.

Advances in CT technology have enhanced the diagnostic value of CCTA, making it a widely used less-invasive imaging modality in the diagnosis of CAD. However, studies based on single centre and multicentre experiences have demonstrated the negative impact of high calcium scores on the specificity and PPV of CCTA.^2–5,24,25^ The specificity of CCTA was reported to be as low as 19% in patients with highly calcified plaques, according to these studies. Our results are consistent with these findings as the specificity of CCTA ranges from 18% to 44% in the CCTA images if no image postprocessing was implemented. The low specificity often leads to unnecessary downstream testing such as ICA or myocardial perfusion imaging, which increases additional cost and radiation dose to patients, thus restricting the clinical application of CCTA. It is therefore important to address this challenge in patients with heavily calcified plaques who undergo CCTA examination.

IR has been increasingly used in cardiac and noncardiac CT imaging because it improves image quality by reducing image noise, while in the meantime significantly lowering the radiation exposure to patients.^26–28^ A number of studies have applied this technique to patients with high calcium scores for assessment of diagnostic value of CCTA, but results are contradictory.^13–19,29^ Renker et al^18^ compared IR with FBP in 55 patients with Agatston scores of more than 400, and they found that the specificity and PPV were increased from 91.2% and 61.1% to 95.5% and 76.9% at per-segment level, 66.7% and 78.9% to 79.2% and 85.7% at per-patient level, corresponding to the CCTA with use of FBP and IR, respectively. Authors also reported that CCTA images using IR significantly lowered the calcification volume compared with those FBP images. This correlates with other studies indicating the significant reduction in calcium score and volume score with use of IR in CCTA compared to FBP images.^15–17^ However, other researchers showed no significant impact of IR on calcium volume or measurement of coronary lumen diameter.^19,29,30^ Thus, it is recommended that applying IR algorithms in CCTA images with high calcification be cautious due to these conflicting results.

Use of image postprocessing technique seems to be an alternative approach when dealing with highly calcified plaques in CCTA images. Despite limited data available in the literature, results of using image postprocessing algorithms in calcified coronary arteries are promising. Tanaka et al^20^ compared subtracted with conventional CCTA images in 11 patients with 55 calcified coronary segments, with ICA as the reference method. At per-segment level, subtraction CCTA shows improvement in specificity which is 59%, compared to the 48.7% with conventional CCTA. The AUC by ROC analysis also shows significant improvement, increasing from 0.741 to 0.905, corresponding to conventional and subtraction CCTA, respectively. The present study was designed according to our previous report which tested a number of image postprocessing algorithms, with “sharpen” reconstruction algorithm confirmed to be most effective in reducing blooming artifacts.^8^ Results of this study further verify the usefulness of CCTA using “sharpen” algorithm in the assessment of calcified coronary arteries. Despite improved diagnostic value being achieved when comparing CCTA_S with CCTA_O or CCTA_OS, the specificity and PPV are still relatively low (<70%), which could generate more ICA or myocardial perfusion imaging. A study by Chow et al^31^ showed significant decrease of normal ICA examinations from 31.5% to 26.8% after CCTA was implemented into clinical practice. However, other studies reported no impact of introduction of CCTA on the number of ICA examinations.^32,33^ A recent study by Zorlak et al^34^ did not show any significant difference in downstream testing between CCTA and SPECT (single-photon emission computed tomography). Due to its limitation of being an anatomical test assessing coronary stenosis, combining CCTA and functional assessment tools has been shown to increase the specificity and positive predictive value, thereby improving the diagnostic workup of patients with significant coronary artery disease.^35,36^ This could be another research direction for assessment of heavily calcified plaques.

The limitations of CCTA overestimation of coronary lumen diameter and stenosis in the presence of severe calcification will not be completely overcome with image postprocessing methods. This has been confirmed in the present study, especially the very low values of AUC by MLD, indicating the low reliability of using MLD for evaluation of coronary lumen diameter due to calcified plaques. Although results in this study have shown significant improvements with use of “sharpen” reconstruction, the diagnostic value of specificity and PPV are still moderate (<70%) in the assessment of highly calcified plaques, indicating the high percentage of false positive results with CCTA. The origin of a calcified plaque may contribute to the moderate performance of CCTA. When a plaque is in close proximity to the coronary surface or intima, it may progress into luminal narrowing, which can be detected on ICA as lumen stenosis, while a plaque arising from the adventitia is likely to be viewed as significant stenosis on CCTA due to false positive results but it may be observed as normal or mild stenosis on ICA.^22^ ICA remains the gold standard for the identification and detection of coronary stenosis, but it fails to provide information about coronary wall, plaque composition, or volume.^37^ Intravascular ultrasound (IVUS) and optical coherence tomography (OCT) are the invasive imaging modalities which allow for quantitative analysis of plaque characteristics, in particular, differentiation of plaque origin by demonstrating the plaque and coronary wall surface changes.^38,39^ Correlation of CCTA findings with IVUS or OCT will enable us to quantitatively assess these calcified plaque features, but this is not the focus of our present study.

Some limitations in this study should be acknowledged. First, the number of eligible patients is relatively low. The small sample size with a limited number of calcified plaques in all 3 coronary arteries limits the generalization of our results. Further studies in a large cohort are needed to verify our findings. Second, no calcium scoring was performed in this study group. Calcium scoring by noncontrast CT is usually performed to assess the coronary calcification which is regarded as a reliable predictor of cardiac events in patients with different age groups. However, recent studies showed no additional benefit to the addition of calcium scoring to contrast-enhanced CT as CCTA can be performed alone with acquisition of more diagnostic information.^40,41^ Third, while this study clearly shows the feasibility of using “sharpen” reconstruction for reduction of blooming artifacts in calcified coronary plaques, this algorithm still needs to be optimized to further improve the diagnostic performance and clinical application of CCTA in clinical practice. Furthermore, other image processing algorithms including some advanced beam hardening algorithms were not applied to our images, thus, the potential benefit of these image processing algorithms cannot be commented. Finally, although our images were acquired with 64- or more slice CT scanners, the spatial resolution (0.5 mm) of the current standard CCTA is inferior to that of the high-definition CT (0.23 mm) which shows improved analysis of calcified plaques for high resolution CCTA.^42^ Further research of combining image processing algorithms with high resolution CT in the analysis of calcified plaques deserves to be investigated.

In conclusion, this study suggests that specificity and PPV of detecting coronary stenosis with CCTA in heavily calcified plaques can be significantly improved with use of “sharpen” reconstruction. However, specificity is still moderate and additional functional imaging may be needed. The enhanced diagnostic performance of CCTA may contribute to reducing the unnecessary invasive or follow-up procedures in patients with extensive calcification in the coronary arteries.
